# Association between primary care physicians’ practice models and referral rates to specialists: A sex-based cross-sectional study

**DOI:** 10.1371/journal.pone.0322175

**Published:** 2025-04-28

**Authors:** Bahram Rahman, David Kirkwood, Glenda Babe, Lauren E. Griffith, David Price, Rebecca H. Correia, Darly Dash, Lauren Lapointe-Shaw, Andrew P. Costa

**Affiliations:** 1 Physician and Provider Services Division, Ministry of Health, Toronto, Ontario, Canada; 2 Department of Health Research Methods, Evidence, and Impact, McMaster University, Hamilton, Ontario, Canada; 3 ICES (formerly known as the Institute for Clinical Evaluative Sciences), Toronto, Ontario, Canada; 4 McMaster Family Health Team, Hamilton, Ontario, Canada; 5 SFU School of Medicine, Simon Fraser University, Burnaby, British Columbia, Canada; 6 Institute of Health Policy, Management and Evaluation, University of Toronto, Toronto, Ontario, Canada; 7 Division of General Internal Medicine and Geriatrics, University Health Network and Sinai Health System, Toronto, Ontario, Canada; 8 Department of Medicine, University of Toronto, Toronto, Ontario, Canada; 9 Centre for Health Economics and Policy Analysis, McMaster University, Hamilton, Ontario, Canada; 10 Department of Medicine, McMaster University, Hamilton, Ontario, Canada; 11 The Research Institute of St. Joe’s Hamilton, St. Joseph’s Healthcare Hamilton, Hamilton, Ontario, Canada; 12 Centre for Integrated Care, St. Joseph’s Health System, Hamilton, Ontario, Canada; Ottawa Hospital, CANADA

## Abstract

Referrals from primary care physicians (PCPs) to specialists are a key function of the primary care system, enabling access to secondary and tertiary health care services. Since the early 2000s, Ontario has implemented substantial primary care practice reforms, however, PCP referral patterns have not been examined since reforms were implemented. We conducted a cross-sectional study in Ontario analyzing PCPs’ referral patterns to specialists from January 1 to December 31, 2019. Data from physician administrative and Ontario Health Insurance Plan (OHIP) billing databases were linked for 9,301 PCPs practicing comprehensive primary care with 11.8 million patients. We calculated referral rates per physician and built a multivariable Poisson regression model stratified by physician sex, recognizing that female and male PCPs practice primary care differently, to examine the association between PCP’s referral rates and their practice model. Subgroup analyses were conducted for medical, surgical, diagnostics and General Practitioner (GP) focused practice specialties. Overall, PCPs in fee-for-service practice models (females: 0.72, 95% CI 0.71–0.72, males 0.71 95% CI 0.71–0.72) and Family Health Groups (females: 0.90, 95% CI 0.90–0.91, males 0.85 95% CI 0.84–0.85) had lower adjusted relative referral rates compared to those in Family Health Teams (FHTs); a finding that was consistent across medical and surgical specialties. Younger, part-time PCPs, those practicing in urban areas, those with larger roster sizes and those affiliated with a large practice group showed higher adjusted referral rates. Female PCPs tended to be younger (average age 47.2 years vs. 54.1 years for males; SMD=0.56), work part-time (32.1% vs. 17.9% for males; SMD=0.33), had a smaller patient roster (average 1,097.8 rostered patients vs. 1,442.1 for males; SMD=0.44), and had higher unadjusted referral rates to specialists compared to male PCPs (32.9 vs. 29.9 per 100 rostered patients). PCPs’ referral patterns in Ontario vary by practice model and PCP’s sex. Future changes to primary care practices should account for their effects on referral volumes to specialists.

## Introduction

Referrals from primary care physicians (PCPs) to specialists are a crucial function of the primary care system. In Ontario, specialists require a referral from a physician or a nurse practitioner to provide publicly funded secondary and tertiary health care services to patients [1,2]. Primary care physicians and specialists combined provide 81% of all medical services in Ontario in a 24-hour period [3]. In 2021/2022 alone more than 8.1 million consultations were provided by specialists referred by PCPs [4].

Since the early 2000s, Ontario’s primary care system has gone under continuous reform, including establishing new funding models for PCPs and specialists. These reforms have drastically changed the composition of the primary care physicians’ workforce (e.g., more female physicians entering medicine) and the ways physician services are funded and practiced [5–8]. One of the main goals of these reforms was to move away from solo fee-for-service practice models more toward team-based care models. Family Health Teams (FHTs), the largest team-based practice model, were introduced in 2005 and, since then, have been gradually expanded [9,10]. PCPs affiliated with a FHT are often paid in a blend of capitation, fee-for-service, pay-for-performance mechanisms or a salary adjusted to the number of patients they service. PCPs in FHTs are supported by a team of publicly funded allied health care professionals, such as nurse practitioners, psychologists, social workers, dietitians, physiotherapists, and others. FHTs were created with the goal of improving outcomes for patients and the health system, including reducing reliance on secondary and tertiary care [10]. Within FHTs, PCPs and allied health care professionals collaborate and provide comprehensive primary health care, primary mental health care, patient education, chronic disease management and preventive care [10]. Despite continued investments in FHTs, there is no consensus on whether they have improved patient outcomes, access to care, or reduced overall health system costs [10–14]. Furthermore, evidence concerning the impact of FHTs compared to other practice models on referrals to specialists is scarce. We sought to examine the referral patterns of PCPs to specialists by practice model, specialties, and their sex. Consistent with reform objectives, we hypothesized that those practicing within the FHT practice model would exhibit lower rates of specialist referrals.

## Methods

### Study design and setting

We conducted a cross-sectional study of PCPs’ referrals to specialists in Ontario between January 1, 2019, to December 31, 2019, with data accessed on August 6, 2023. Ontario has 16.2 million residents and is the most populous province in Canada [15]. Medically necessary physician services, including primary care and specialist care, are publicly funded. In 2022, there were 35,320 physicians in Ontario, of which 17,416 worked in general practice and family medicine, including comprehensive primary care services; 17,904 physicians practiced other specialties [16]. PCPs practice in one of several practice models including Family Health Groups (mix of fee-for-service, bonuses, and premiums), capitated non-team models (capitation, bonuses, premiums, and fee-for-service without interdisciplinary teams), Family Health Teams (allied health care professionals support with capitation bonuses, premiums, and fee-for-service or salaried payments), solo fee-for-service, and other patient enrollment or group-based salaried models [17]. Specialists tend to practice as solo providers, providers in teams in the community or in a hospital setting [8].

### Data sources and study population

**Fig 1:**
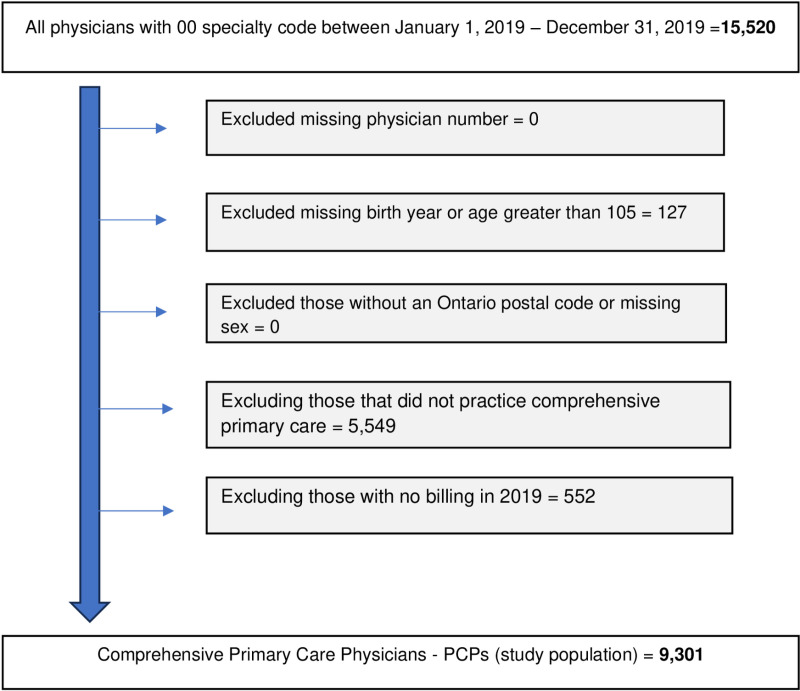
Inclusion and exclusion criteria.

We linked multiple health administrative datasets at ICES (formerly known as the Institute for Clinical Evaluative Sciences). ICES is an independent, non-profit research institute whose legal status under Ontario’s health information privacy law allows it to collect and analyze health care and demographic data without consent for health system evaluation and improvement. We included all PCPs who practiced comprehensive primary care services actively between January 1, 2019, to December 31, 2019. Comprehensive primary care includes a broad spectrum of health services to meet the full range of patient’s health-related needs and arrange for the resources to deal with them (S1 Table includes billing codes used to identify comprehensive primary care physicians) [18]. We used data prior to the COVID-19 pandemic to reduce any risks of distortions caused by the pandemic lockdowns and post pandemic recovery. PCPs’ characteristics were obtained from the ICES Physician Database (IPDB). Ontario Health Insurance Plan (OHIP) billing data were used for information about PCPs’ patients and practice characteristics. These datasets were linked with unique coded identifiers and were analyzed at ICES. Fig 1 presents the complete exclusion criteria of the study population. The data used in this study, consisting of secondary and deidentified information accessed through ICES, is authorized under *Section 45 of Ontario’s Personal Health Information Protection Act, 2004* and did not require approval from an ethics review board.

### Dependent variable

We calculated the rate of referrals to specialists per 100 rostered patients (included both formally enrolled and virtually rostered). We included only those specialist referrals that were requested by patient’s rostered PCP and completed (rendered a consultation) within the 12-month study period. We excluded follow-up visits requested by the specialist, referrals from a specialist to another specialist, referrals from PCPs other than the patients’ rostering PCP (e.g., walk-in clinic physician), and consults that were hospital-based or performed in an emergency department.

### Independent variables

The main independent variable of interest was the PCP’s primary care practice model (categorized). We included rostered patients’ characteristics, such as the mean age of rostered patients, the proportion of rostered patients that were female and the proportion of rostered patients with twice the population’s average level of medical complexity, using the CIHI Population Grouper Methodology [19]. Physicians’ characteristics such as age, sex, roster size (categorized), practice distance (Kilometers) from an academic hospital (categorized), full-time equivalency (categorized), community size as a proxy for rurality (categorized) and PCP’s group size (categorized) were also included. (S2 Table includes a complete description of data sources for each variable.)

### Statistical analysis

Means and standard deviations (for continuous variables, after testing for normal distribution) and proportions (for categorical variables) were calculated separately for female and male PCPs in recognition of previously reported differences in their practice patterns [20–25]. We calculated standardized differences to compare practice patterns between female and male PCPs with differences greater than 10% considered meaningful [26]. We used a multivariable Poisson regression model stratified by physician’s sex to estimate unadjusted and adjusted rate ratios for each dependent variable. We used a generalized linear mixed model (GLMM) with a Poisson distribution and a log link with an exchangeable correlation structure to account for clustering at the physician’s group level. An offset term of the log of the total number of rostered patients for the physicians divided by 100 was used to obtain the rate ratio per 100 rostered patients. The adjusted model included the following variables: mean age of rostered patients, percent of rostered female patients, percent of rostered patients twice or more complex than the provincial average, physician’s age, total roster size, distance of the practice from an academic hospital, physician’s FTE, community size and the PCP’s group size. These variables were selected based on existing evidence of their impact on PCP’s practice patterns and policy relevance. Subgroup analyses were conducted for different specialties: surgical, medical, diagnostics, and GP-focused practice (S3 Table includes physician specialties classification). We reported the results as adjusted rate ratios (RRs) with 95% confidence intervals (CIs). We considered a 2-tailed p-value of less than 0.05 significant. We conducted all analyses using SAS Enterprise Guide software, version 8.3 in the ICES Remote Access Environment (RAE).

## Results

We identified 9,301 PCPs with 11.8 million rostered patients (Fig 1). There were 4,653 female PCPs and 4,648 male PCPs. Female PCPs tended to be younger (average age 47.2 years vs. 54.1 years for males; SMD=0.56), work part-time (32.1% vs. 17.9% for males; SMD=0.33), and had smaller patient roster (average 1,097.8 rostered patients vs. 1,442.1 for males; SMD=0.44). Female PCPs also tended to refer more often to other female specialists (32.5% vs. 25.4%; SMD=0.16). We did not observe meaningful differences between male and female PCPs regarding their affiliations to primary care practice models and the size of their group (Table 1).

**Table 1:** PCPs practice characteristics, Ontario, January 1, 2019, to December 31, 2019.

Practice Characteristics	All PCPs	Female PCPs	Male PCPs	Standardized Difference
	N=9,301	N=4,653	N=4,648	
**Practice model type** ^*^				
Family Health Groups - n (%)	2,370 (25.5%)	1,115 (24.0%)	1,255 (27.0%)	0.07
Capitated non-Teams - n (%)	2,775 (29.8%)	1,417 (30.5%)	1,358 (29.2%)	0.03
Family Health Teams - n (%)	2,484 (26.7%)	1,330 (28.6%)	1,154 (24.8%)	0.08
Solo FFS - n (%)	1,332 (14.3%)	667 (14.3%)	665 (14.3%)	0.00
Other PEM models - n (%)	340 (3.7%)	124 (2.7%)	216 (4.6%)	0.11
**Total number of rostered patients - Mean (SD)**	1,269.83 (803.08)	1,097.76 (705.35)	1,442.09 (856.35)	0.44
**PCPs age - Mean (SD)**	50.63 (12.79)	47.19 (11.50)	54.07 (13.09)	0.56
**PCPs age – categories**				
<40 yrs.- n (%)	2,319 (24.9%)	1,490 (32.0%)	829 (17.8%)	0.33
40-49 yrs.- n (%)	2,049 (22.0%)	1,216 (26.1%)	833 (17.9%)	0.20
50-59 - n (%)	2,417 (26.0%)	1,151 (24.7%)	1,266 (27.2%)	0.06
60-69 - n (%)	1,836 (19.7%)	680 (14.6%)	1,156 (24.9%)	0.26
>70 - n (%)	680 (7.3%)	116 (2.5%)	564 (12.1%)	0.38
**Community size**				
Large Urban- n (%)	4,375 (47.0%)	2,205 (47.4%)	2,170 (46.7%)	0.01
Medium Urban - n (%)	1,680 (18.1%)	924 (19.9%)	756 (16.3%)	0.09
Small Urban- n (%)	1,837 (19.8%)	888 (19.1%)	949 (20.4%)	0.03
Rural- n (%)	610 (6.6%)	267 (5.7%)	343 (7.4%)	0.07
Remote - n (%)	601 (6.5%)	267 (5.7%)	334 (7.2%)	0.06
Missing - n (%)	198 (2.1%)	102 (2.2%)	96 (2.1%)	0.01
**PCPs FTE (Quartile)**				
Q1 (Equal or lower than 0.8766) - n (%)	2,326 (25.0%)	1,494 (32.1%)	832 (17.9%)	0.33
Q2 (0.8766–1) - n (%)	2,703 (29.1%)	1,631 (35.1%)	1,072 (23.1%)	0.27
Q3 (1–1.271) - n (%)	1,946 (20.9%)	888 (19.1%)	1,058 (22.8%)	0.09
Q4 (Equal or greater than 1.271) - n (%)	2,326 (25.0%)	640 (13.8%)	1,686 (36.3%)	0.54
**Panel size (Quartile)**				
Q1 (<800) - n (%)	2,506 (26.9%)	1,488 (32.0%)	1,018 (21.9%)	0.23
Q2 (800–1,299) - n (%)	2,687 (28.9%)	1,615 (34.7%)	1,072 (23.1%)	0.26
Q3 (1,300–2,399) - n (%)	3,388 (36.4%)	1,361 (29.2%)	2,027 (43.6%)	0.30
Q4 (>2,400) - n (%)	720 (7.7%)	189 (4.1%)	531 (11.4%)	0.28
**Practice distance from an academic hospital (Kilometer)**				
Greater (>10 km) - n (%)	6,074 (65.3%)	2,886 (62.0%)	3,188 (68.6%)	0.14
5-10 km - n (%)	1,290 (13.9%)	657 (14.1%)	633 (13.6%)	0.02
Lower (5 km) - n (%)	1,937 (20.8%)	1,110 (23.9%)	827 (17.8%)	0.15
**PCPs group size**				
1 physician - n (%)	294 (3.2%)	146 (3.1%)	148 (3.2%)	0.00
2 physicians - n (%)	290 (3.1%)	135 (2.9%)	155 (3.3%)	0.03
3-4 physicians - n (%)	613 (6.6%)	289 (6.2%)	324 (7.0%)	0.03
>5 physicians - n (%)	8,104 (87.1%)	4,083 (87.7%)	4,021 (86.5%)	0.04
**Sex of specialist referred to – n (%)**				
Female - n (%)	1,056,907 (28.7%)	547,278 (32.5%)	509,629 (25.4%)	0.16
Male - n (%)	2,630,140 (71.3%)	1,135,570 (67.5%)	1,494,570 (74.6%)	
**Total visits per rostered patient – mean (SD)**	3.6 (1.9)	3.3 (1.8)	3.8 (1.9)	0.26

* Family Health Group where physicians are paid a mix of fee-for-service along with bonuses and premiums. Capitated non-Team includes models, i.e., Family Health Organization and Family Health Network where physicians are paid a mix of capitation payment, bonuses, premiums, and fee-for-service but they are not part of a Family Health Team (FHT). FHTs are interdisciplinary models of care, where physician can be paid through capitation with bonuses, premiums, and fee-for-service or salaried mechanisms. Solo FFS: Patients are not formally part of an enrolment model but receive care from a regular primary care physician who is paid purely fee-for-service. Other PEM models include smaller specialized patient enrolment models.

Female PCPs also had a higher proportion of rostered female patients (60.5% vs. 44.7% for males; SMD=0.32) and on average rostered younger patients. We did not observe a meaningful difference between male and female PCPs regarding the complexity of their rostered patients (Table 2).

**Table 2:** Patients’ characteristics, Ontario, January 1, 2019, to December 31, 2019.

Patient Characteristics	All Rostered Patients	Female PCPs	Male PCPs	Standardized Difference
	N=11,810,698	N=5,107,873	N=6,702,825	
**Patients age - Mean (SD)**	41.05 (8.25)	38.97 (7.77)	43.13 (8.19)	0.52
**Patients sex**				
Female - n (%)	6,140,036 (52.0%)	3,108,390 (60.9%)	3,031,646 (45.2%)	0.32
Male - n (%)	5,670,662 (48.0%)	1,999,483 (39.1%)	3,671,179 (54.8%)	
**Patients with twice the complexity relative to the average population** ^**^				
No - n (%)	10,188,586 (86.3%)	4,425,218 (86.6%)	5,763,368 (86.0%,)	0.02
Yes - n (%)	1,622,112 (13.7%)	682,655 (13.4%)	939,457 (14.0%)	

** Based on the CIHI’s Population Grouping Methodology.

PCPs in FHTs had a lower unadjusted referral rate per 100 rostered patients to any specialist than those in Capitated non-Team (32.6 vs 34.7; p-value <0.0001); however, they had a higher rate than other models (Table 3). This finding was consistent across all subspecialties. (S4 Table includes additional descriptive analysis by primary care practice model).

**Table 3:** Referral rates per 100 rostered patients by primary care practice model, Ontario, January 1, 2019, to December 31, 2019.

	Family Health Group	Capitated non-Team	Family Health Team	Solo FFS	Other PEM Models
**Referrals rate – all specialties**	28.3	34.7	32.6	20.2	30.2
**Diagnostic referrals**	0.1	0.1	0.1	0.00	0.01
**GP focused practice referrals**	2.1	2.6	2.6	1.5	2.1
**Medical referrals**	14.3	17.1	15.1	10.1	14.9
**Surgical referrals**	11.7	14.8	14.7	8.6	12.9

Note: All comparisons P <.0001.

Overall, female PCPs had a higher unadjusted referral rate per 100 rostered patients to any specialist compared to male PCPs (32.9 vs. 29.9; p-value <0.0001) (Table 4).

**Table 4:** Referral rates per 100 rostered patients by PCP’s sex, Ontario, January 1, 2019, to December 31, 2019.

	All PCPs	Female PCPs	Male PCPs	Absolute Difference
**Referrals – all specialties**	31.2	32.9	29.9	3.0
**Diagnostic referrals**	0.1	0.1	0.1	0.0
**GP focused practice referrals**	2.4	2.6	2.2	0.4
**Medical referrals**	15.3	16.5	14.3	2.2
**Surgical referrals**	13.4	13.7	13.3	0.4

Note: All comparisons P <.0001.

For both sexes, the top specialties for which a referral was made were general surgery, dermatology, OB/GYN, internal medicine, and GP-focused practice (Table 5).

**Table 5:** Top ten specialties that PCPs made a referral to (count and percent), Ontario, January 1, 2019, to December 31, 2019.

	All PCPs	Female PCPs	Male PCPs
**General Surgery -** n (%)	371,071 (10.1%)	155,200 (9.2%)	215,871 (10.8%)
**Dermatology -** n (%)	340,156 (9.2%)	166,990 (9.9%)	173,166 (8.6%)
**OB/GYN -** n (%)	304,702 (8.3%)	157,147 (9.3%)	147,555 (7.4%)
**Internal Medicine -** n (%)	301,120 (8.2%)	125,439 (7.5%)	175,681 (8.8%)
**GP focused practice -** n (%)	280,228 (7.6%)	132,788 (7.9%)	147,440 (7.4%)
**Orthopedic Surgery -** n (%)	250,593 (6.8%)	104,040 (6.2%)	146,553 (7.3%)
**Otolaryngology -** n (%)	241,321 (6.5%)	109,717 (6.5%)	131,604 (6.6%)
**Cardiology -** n (%)	200,295 (5.4%)	86,356 (5.1%)	113,939 (5.7%)
**Gastroenterology -** n (%)	188,424 (5.1%)	88,989 (5.3%)	99,435 (5.0%)
**Urology -** n (%)	162,245 (4.4%)	62,588 (3.7%)	99,657 (5.0%)

PCPs in Family Health Group (FHG) practices showed a lower referral rate to specialists compared to those in FHT practices. Both female and male PCPs in FHG practices had lower referral rates (female PCPs: RR=0.90, 95% CI=0.90–0.91 vs. male PCPs: RR=0.85, 95% CI=0.84–0.85) compared to PCPs in FHT practices. Similarly, both female and male PCPs in solo FFS practices had lower referral rates compared to those in FHTs (female PCPs: RR=0.72, 95% CI: 0.71–0.72 vs. male PCPs: RR=0.71, 95% CI=0.71–0.72). There were no statistically significant differences observed between PCPs in Capitated non-Team practices compared to FHT practices (Table 6). Younger and part-time PCPs had a higher rate of referrals to specialists compared to their older counterparts and those working full-time or more. Also, PCPs practicing in large or medium-sized urban areas had a higher specialist referral rate compared to those in rural areas (Table 6).

**Table 6:** Adjusted relative risk of specialist referrals, stratified by PCP’s sex, Ontario, January 1, 2019, to December 31, 2019.

Practice Characteristics	Female PCPs RR (95% CI)	Male PCPs RR (95% CI)
**Practice model type** ^***^		
Family Health Teams (ref)	(ref)	(ref)
Family Health Groups	0.90 (0.90-0.91)	0.85 (0.84-0.85)
Capitated non-Teams	1.03 (1.03-1.04)	1.00 (0.99-1.00)
Solo FFS	0.72 (0.71-0.72)	0.71 (0.71-0.72)
Other PEM models	1.02 (1.01-1.03)	0.85 (0.84-0.85)
**Patients age (mean difference, 1 year)**	1.01 (1.01-1.02)	1.02 (1.01-1.02)
**Patients sex (mean % of rostered female patients)**	1.01 (1.01-1.01)	1.00 (1.00-1.01)
**Patients’ complexity (twice the complexity relative to the average population)**		
No	(ref)	(ref)
Yes	1.04 (1.04-1.04)	1.05 (1.04-1.05)
**PCPs age – categories**		
<40 yrs. (ref)	(ref)	(ref)
40-49 yrs.	1.09 (1.08-1.09)	0.98 (0.97-0.98)
50-59 yrs.	1.03 (1.02-1.03)	0.96 (0.96-0.97)
60-69 yrs.	1.00 (0.99-1.00)	0.89 (0.89-0.90)
>70 yrs.	0.88 (0.87-0.89)	0.80 (0.80-0.81)
**Community size**		
Large Urban	1.18 (1.17-1.19)	1.16 (1.15-1.17)
Medium Urban	1.18 (1.17-1.19)	1.17 (1.16-1.18)
Small Urban	1.17 (1.16-1.19)	1.17 (1.17-1.18)
Rural (ref)	(ref)	(ref)
Remote	1.02 (1.01-1.03)	0.96 (0.95-0.97)
Missing	1.12 (1.10-1.14)	1.09 (1.07-1.10)
**PCPs FTE (Quartile)**		
Q1 (Equal or lower than 0.8766) (ref)	(ref)	(ref)
Q2 (0.8766–1)	0.98 (0.98-0.99)	1.02 (1.02-1.03)
Q3 (1–1.271)	0.93 (0.93-0.94)	0.99 (0.98-0.99)
Q4 (Equal or greater than 1.271)	0.90 (0.90-0.91)	0.98 (0.97-0.99)
**Roster size (Quartile)**		
Q1 (<800) (ref)	(ref)	(ref)
Q2 (800–1,299)	1.10 (1.09-1.10)	1.18 (1.17-1.19)
Q3 (1,300–2,399)	1.13 (1.12-1.14)	1.26 (1.25-1.27)
Q4 (>2,400) - n	1.10 (1.09-1.11)	1.32 (1.30-1.33)
**Practice distance from an academic hospital (Kilometer)**		
Lower (5 km) (ref)	(ref)	(ref)
5-10 km	1.02 (1.01-1.02)	(1.01-1.02)
Higher (>10) km	1.03 (1.03-1.04)	1.06 (1.05-1.06)
**PCPs group size**		
1 physician (ref)	(ref)	(ref)
2 physicians	1.12 (0.95-1.32)	1.01 (0.85-1.20)
3-4 physicians	1.31 (1.13-1.52)	1.15 (0.99-1.35)
5+ physicians	1.53 (1.33-1.75)	1.32 (1.14-1.52)

*** Family Health Group where physicians are paid a mix of fee-for-service along with bonuses and premiums. Capitated non-Team includes models, i.e., Family Health Organization and Family Health Network where physicians are paid a mix of capitation payment, bonuses, premiums, and fee-for-service but they are not part of a Family Health Team (FHT). FHTs are interdisciplinary models of care, where physician can be paid through capitation with bonuses, premiums, and fee-for-service or salaried mechanisms. Solo FFS: Patients are not formally part of an enrolment model but receive care from a regular primary care physician who is paid purely fee-for-service. Other PEM models include smaller specialized patient enrolment models.

Female and male PCPs in FHG and solo FFS practices had lower adjusted referral rates to medical and surgical specialties compared to those in FHT practices. PCPs in solo FFS practices had lower adjusted referral rates for diagnostic specialties compared to FHT practices. However, aside from solo FFS practices, all other practice models demonstrated higher adjusted referral rates to GP-focused specialties compared to FHTs (S1 Fig includes adjusted association of referral to different specialties).

## Discussion

Our findings demonstrate differences in referral patterns between PCP practice models and PCPs’ sex. PCPs affiliated with fee-for-service practice models (FHG and solo FFS) had lower referral rates compared to those in FHT practices. Our finding aligns with previous studies that found an association between primary care model type and specialist referral at the population level where patients receiving care from a fee-for-service model had a lower referral rates compared to those in capitated or interdisciplinary models [27,28]. PCPs in FFS practice models have a financial incentive to provide more services within their practice instead of referring to specialists compared to PCPs in capitated and salaried models where most of their income is guaranteed regardless of their referral patterns. FHT practices also have access to more resources and support for their rostered patients, which could induce demand for more medical services that, in some cases, might not be required (e.g., a physiotherapist may suggest referral to an orthopedic specialist) [29–33]. There could also be unexplained variability in PCP’s decision to refer their rostered patients to one or more specialties [25].

We described PCPs’ specialist referral patterns and evaluated variations between female and male PCPs, without asserting the appropriateness of these referrals. Our study addresses key limitations of previous research, such as being conducted prior to primary care reform, relying on self-reported data, focusing solely on referrals from electronic consultation platforms, and not accounting for shifts in the physician workforce such as the growth in GP-focused specialties [34–37]. GP focused practices dedicate most of their clinic to one area such as sports medicine, pain medicine, sleep medicine, anesthesiology, dermatology and others [38].

We found differences in referral patterns between female and male PCPs, including their age, workload, and referrals made to different specialties. Our findings support an emerging body of evidence on variations between female and male PCP practice in primary care, including differences in workload and working hours [20,21], preference to refer to specialists of their own sex [21], and the impact of their practice patterns on their billing practices [24,25]. We found higher unadjusted referral rates for female PCPs compared to their male counterparts for all specialists, also reported by Liddy et.al (2014) [27] and across different specialties.

Our analysis also showed differences in referral rates across specialties, with PCPs in different practice models exhibiting distinct referral patterns. For instance, PCPs in FHG and solo FFS practices showed lower referral rates to medical and surgical specialties compared to those in FHT practices. On the other hand, PCPs in FHT practices had lower referral rates to GP focused practices. The lower referral rates to GP-focused practices may be explained by PCPs in capitated payment models, including those in FHT practices, being hesitant to refer their rostered patients to other GPs due to concerns about potential financial impacts on the bonus they receive for providing comprehensive primary care services within their rostering group. PCPs in FHT practices could also refer their rostered patients to allied health care services within their team (e.g., mental health, physiotherapy) instead of GP focused practices.

### Policy implications

Efforts to expand FHTs need to consider the potential health system burden of more specialist referrals and could introduce interventions (i.e., incentives, quality improvement supports and accountabilities) to reduce unnecessary referrals. Policymakers could consider reforming the FHT practice model with clear performance goals, including reducing specialists and high-cost services as intended during the development of FHTs. Secondly, policymakers should carefully assess the impact of GP-focused practices on comprehensive primary care before expanding these programs. Such programs could strain on the primary care system by diverting funding and human resources away from comprehensive care. Finally, our findings highlight the need for ongoing monitoring and evaluation of referral patterns within primary care practices to identify areas for improvement and ensure equitable and timely access to specialist services. This could involve the development of standardized referral guidelines, decision support tools and quality indicators to monitor referral appropriateness and efficiency.

### Limitations

The cross-sectional design of the study limits our ability to establish causal relationships between PCP characteristics and referral patterns. Longitudinal studies are needed to further explore factors influencing referral decisions over time, especially whether the PCPs changed behavior with the availability of virtual primary care and specialty services [39,40] and reported administrative burdens experienced by PCPs [41]. Our analysis relied on administrative data, which may not capture all relevant variables influencing referral patterns, such as clinical factors, patient preferences, referral wait-times, and PCP attitudes. Our data also excluded referrals that were not completed within the one-year period, which may underrepresent overall referral volumes. However, we expect this impact to be small and unlikely to change the overall conclusion of our findings. Further research could incorporate contextual explanatory methods providing deeper insights into the complex drivers of primary care physicians’ referral decision-making. Our study focused solely on referrals within the Ontario health care system, which may not be generalizable to other jurisdictions where a referral is not needed to access specialist care or they have a different configuration of team-based care models.

## Conclusion

Primary care physicians’ referral rates in Ontario vary by practice model. Contrary to their policy goals, FHTs showed comparable rates to Capitated non-Team and higher rates compared to fee-for-service models. These findings underscore the importance anticipating referral volumes to specialists as practice models and provider characteristics change.

## Supporting information

S1 Table Comprehensive primary care codes. (DOCX)

S2 Table Description of data sources and variables. (DOCX)

S3 Table Physician specialties classification. (DOCX)

S4 Table PCPs’ and their patients’ characteristics by practice model, Ontario, January 1, 2019, to December 31, 2019. (DOCX)

S5 Table Unadjusted relative risk of specialist referrals, stratified by PCP’s sex, Ontario, January 1, 2019, to December 31, 2019. (DOCX)

S6 File A brief description of Family Health Teams. (DOCX)

S7 File STROBE Statement—Checklist of items that should be included in reports of cross-sectional studies. (DOCX)

S1 Fig Adjusted association of referrals to different specialties, stratified by PCP’s sex, Ontario, January 1, 2019, to December 31, 2019. (DOCX)
