# Supplementary material for: Association between primary care physicians’ practice models and referral rates to specialists: A sex-based cross-sectional study
Source: PLoS One. 2025 Apr 28;20(4):e0322175. doi: 10.1371/journal.pone.0322175 (PMC12036902; doi:10.1371/journal.pone.0322175)
Supplement: S1 Table — (DOCX) [file pone.0322175.s001.docx]

**S1 Table.** Comprehensive primary care codes.

| **OHIP Codes** | **Description** |
| --- | --- |
| A001 | Minor assessment |
| A002 | Enhanced 18 month well baby visit |
| A003 | General assessment |
| A007 | Intermediate assessment |
| A903 | Preoperative assessment |
| E075 | Geriatric general assessment |
| G212 | Allergy injection alone |
| G271 | Anticoagulant supervision |
| G372 | Injection, with visit |
| G373 | Injection, sole reason |
| G365 | Papanicolaou test |
| G538 | Immunization, with visit |
| G539 | Immunization, sole reason |
| G590 | Influenza immunization, with visit |
| G591 | Influenza immunization, sole reason |
| K005 | Primary mental health care |
| K013 | Counselling, individual care |
| K017 | Annual health exam |
| P004 | Minor prenatal assessment |
| K130 | Periodic health visit, adolescent |
| K131 | Periodic health visit, adult aged 18 to 64 inclusive |
| K132 | Periodic health visit, adult 65 years of age and older |
| K030 | Diabetic management |
| K080 | Minor assessment, Covid, virtual |
| K081 | Intermediate assessment, Covid, virtual |
| K082 | Primary mental health care, Covid, virtual |
| A261 | Minor assessment, pediatric |
| A268 | Pediatrics, enhanced 18 months well baby visit |
| A267 | Annual health exam, child 2–11 years |
| K269 | Annual adolescent health exam |

**Reference:**

Schultz SE, Glazier RH. Identification of physicians providing comprehensive primary care in Ontario: A retrospective analysis using linked administrative data. CMAJ Open. 2017;5(4).
