# Supplementary material for: Association between primary care physicians’ practice models and referral rates to specialists: A sex-based cross-sectional study
Source: PLoS One. 2025 Apr 28;20(4):e0322175. doi: 10.1371/journal.pone.0322175 (PMC12036902; doi:10.1371/journal.pone.0322175)
Supplement: S2 Table — (DOCX) [file pone.0322175.s002.docx]

**S2 Table.** Description of data sources and variables.

| **Variables** | **Database** | **Description** |
| --- | --- | --- |
| Patient age | Registered Persons Database (RPBD) | RPDB is a personal information bank that contains information on all Ontario residents registered for the Ontario Health Insurance Plan (OHIP) and Ontario Drug Benefit (ODB). |
| Patient sex |  |  |
| Patient complexity | CIHI Grouping Methodology | The Population Grouping Methodology (POP Grouper) using a case-mix classification case-mix classification to profile each person in the population using person level clinical information. It was developed by the Canadian Institute for Health Information (CIHI) to help meet the needs of Canada’s health care systems. |
| PCP’s age | ICES Physician Database (IPDB) | IPDB contains yearly information about all physicians in Ontario. Its potential uses include physician profiling, predicting physician behaviour, measuring physician supply, and many others. |
| PCP’s sex |  |  |
| PCP’s FTE |  |  |
| Roster size |  |  |
| Practice distance |  |  |
| Payment model |  |  |
| PCP’s community size | Census – 2016 Ontario | Statistics Canada census data provides comprehensive demographic, social, and economic information about the Canadian population. |
| PCP’s practice model | ICES Primary Care Population (PCPOP) | PCPOP is a population-level dataset that includes all people in Ontario who are deemed alive and eligible at a given point in time. All indicators are as of the index date, with various look-back periods and an extensive list of primary care related indicators. |
| PCP’s group size | Client Agency Program Enrolment (CAPE) | CAPE contains a list of patients enrolled with a specific primary care enrolment physician model, including the patient's enrolment status (active or inactive), and the group they are associated with. |
| Referral rate | Ontario Health Insurance Plan Claims Database (OHIP) | OHIP contains most claims paid for by the Ontario Health Insurance Plan. The data cover all health care providers who can claim under OHIP (this includes physicians, groups, laboratories, and out-of-province providers). |
| Note: The dataset from this study is held securely in coded form at ICES (formerly known as the Institute for Clinical Evaluative Sciences). | | |
