# Supplementary material for: Association between primary care physicians’ practice models and referral rates to specialists: A sex-based cross-sectional study
Source: PLoS One. 2025 Apr 28;20(4):e0322175. doi: 10.1371/journal.pone.0322175 (PMC12036902; doi:10.1371/journal.pone.0322175)
Supplement: S3 Table — (DOCX) [file pone.0322175.s003.docx]

**S3 Table.** Physician specialties classification.

| **Code** | **Specialty description** | **Specialty category** | **Assembly** |
| --- | --- | --- | --- |
| 28 | Pathology, Microbiology, Clinical Biochemistry | Other specialties | Diagnostic |
| 33 | Diagnostic Radiology | Imaging | Diagnostic |
| 63 | Nuclear Medicine | Imaging | Diagnostic |
| 00 | Family Practice & General Practice | Family & General Practice | GP focused |
| 02 | Dermatology | Medical non-procedural | Medical |
| 05 | Community Medicine | Other specialties | Medical |
| 07 | Geriatrics | Medical non-procedural | Medical |
| 13 | Internal Medicine | Medical non-procedural | Medical |
| 15 | Endocrinology | Medical non-procedural | Medical |
| 16 | Nephrology | Medical procedural | Medical |
| 18 | Neurology | Medical non-procedural | Medical |
| 19 | Psychiatry | Medical non-procedural | Medical |
| 22 | Genetics | Other specialties | Medical |
| 26 | Pediatrics | Medical non-procedural | Medical |
| 31 | Physical Medicine | Medical non-procedural | Medical |
| 34 | Therapeutic Radiology | Medical procedural | Medical |
| 41 | Gastroenterology | Medical procedural | Medical |
| 44 | Medical Oncology | Medical non-procedural | Medical |
| 46 | Infectious Disease | Other specialties | Medical |
| 47 | Respiratory Disease | Medical procedural | Medical |
| 48 | Rheumatology | Medical non-procedural | Medical |
| 60 | Cardiology | Medical procedural | Medical |
| 61 | Hematology | Medical non-procedural | Medical |
| 62 | Clinical Immunology | Medical non-procedural | Medical |
| 11 | Critical Care | Medical non-procedural | Medical |
| 03 | General Surgery | Surgical | Surgical |
| 04 | Neurosurgery | Surgical | Surgical |
| 06 | Orthopedic Surgery | Surgical | Surgical |
| 08 | Plastic Surgery | Surgical | Surgical |
| 09 | Cardiothoracic Surgery | Surgical | Surgical |
| 17 | Vascular Surgery | Surgical | Surgical |
| 20 | Obstetrics & Gynecology | Surgical | Surgical |
| 23 | Ophthalmology | Surgical | Surgical |
| 24 | Otolaryngology | Surgical | Surgical |
| 35 | Urology | Surgical | Surgical |
| 64 | Thoracic Surgery | Surgical | Surgical |
