# Supplementary material for: Association between primary care physicians’ practice models and referral rates to specialists: A sex-based cross-sectional study
Source: PLoS One. 2025 Apr 28;20(4):e0322175. doi: 10.1371/journal.pone.0322175 (PMC12036902; doi:10.1371/journal.pone.0322175)
Supplement: S4 Table — (DOCX) [file pone.0322175.s004.docx]

**S4 Table.** PCPs’ and their patients’ characteristics by practice model, Ontario, January 1, 2019, to December 31, 2019.

**Table S4.A:** PCPs’ characteristics by practice model, Ontario, January 1, 2019, to December 31, 2019.

| **Practice** **Characteristics** | **Total PCPs** | **Family Health Group** | **Capitated non-Team** | **Family Health Team** | **Solo FFS** | **Other PEM Models** |
| --- | --- | --- | --- | --- | --- | --- |
|  | N=9,301 | n=2,370 | n=2,775 | n=2,484 | n=1,332 | n=340 |
| **PCPs sex** |  |  |  |  |  |  |
| Female - n (%) | 4,653 (50.0%) | 1,115 (47.0%) | 1,417 (51.1%) | 1,330 (53.5%) | 667 (50.1%) | 124 (36.5%) |
| Male - n (%) | 4,648 (50.0%) | 1,255 (53.0%) | 1,358 (48.9%) | 1,154 (46.5%) | 665 (49.9%) | 216 (63.5%) |
| **Total number of rostered patients - Mean (SD)** | 1,269.83 (803.08) | 1574.22 (948.33) | 1406.27 (639.90) | 1253.42 (607.07) | 441.03 (544.10) | 1401.31 (823.00) |
| **PCPs age - Mean (SD)** | 50.63 (12.79) | 52.86 (12.40) | 50.99 (12.41) | 48.51 (11.92) | 48.84 (14.89) | 54.66 (11.90) |
| **PCPs age – categories** |  |  |  |  |  |  |
| <40 yrs.- n (%) | 2,319 (24.9%) | 408 (17.2%) | 654 (23.6%) | 741 (29.8%) | 476 (35.7%) | 40 (11.8%) |
| 40-49 yrs.- n (%) | 2,049 (22.0%) | 527 (22.2%) | 598 (21.5%) | 607 (24.4%) | 248 (18.6%) | 69 (20.3%) |
| 50-59 - n (%) | 2,417 (26.0%) | 707 (29.8%) | 745 (26.8%) | 608 (24.5%) | 246 (18.5%) | 111 (32.6%) |
| 60-69 - n (%) | 1,836 (19.7%) | 498 (21.0%) | 592 (21.3%) | 435 (17.5%) | 228 (17.1%) | 83 (24.4%) |
| >70 - n (%) | 680 (7.3%) | 230 (9.7%) | 186 (6.7%) | 93 (3.7%) | 134 (10.1%) | 37 (10.9%) |
| **Community size** |  |  |  |  |  |  |
| Large Urban- n (%) | 4,375 (47.0%) | 4,375 (47.0%) | 1,654 (69.8%) | 1,216 (43.8%) | 646 (26.0%) | 655 (49.2%) |
| Medium Urban - n (%) | 1,680 (18.1%) | 1,680 (18.1%) | 307 (13.0%) | 629 (22.7%) | 455 (18.3%) | 246 (18.5%) |
| Small Urban- n (%) | 1,837 (19.8%) | 1,837 (19.8%) | 324 (13.7%) | 606 (21.8%) | 596 (24.0%) | 260 (19.5%) |
| Rural- n (%) | 610 (6.6%) | 610 (6.6%) | 25 (1.1%) | 125 (4.5%) | 398 (16.0%) | 51 (3.8%) |
| Remote - n (%) | 601 (6.5%) | 601 (6.5%) | 31 (1.3%) | 134 (4.8%) | 326 (13.1%) | *80-84 |
| Missing - n (%) | 198 (2.1%) | 198 (2.1%) | 29 (1.2%) | 65 (2.3%) | 63 (2.5%) | *36-40 |
| **PCPs FTE (Quartile)** |  |  |  |  |  |  |
| Q1 (Equal or lower than 0.8766) - n (%) | 2,326 (25.0%) | 593 (25.0%) | 280 (10.1%) | 451 (18.2%) | 919 (69.0%) | 83 (24.4%) |
| Q2 (0.8766 - 1) - n (%) | 2,703 (29.1%) | 726 (30.6%) | 878 (31.6%) | 737 (29.7%) | 241 (18.1%) | 121 (35.6%) |
| Q3 (1 - 1.271) - n (%) | 1,946 (20.9%) | 444 (18.7%) | 718 (25.9%) | 625 (25.2%) | 97 (7.3%) | 62 (18.2%) |
| Q4 (Equal or greater than 1.271) - n (%) | 2,326 (25.0%) | 607 (25.6%) | 899 (32.4%) | 671 (27.0%) | 75 (5.6%) | 74 (21.8%) |
| **Roster size (Quartile)** |  |  |  |  |  |  |
| Q1 (<800) - n (%) | 2,506 (26.9%) | 457 (19.3%) | 365 (13.2%) | 531 (21.4%) | 1,072 (80.5%) | 81 (23.8%) |
| Q2 (800-1,299) - n (%) | 2,687 (28.9%) | 601 (25.4%) | 959 (34.6%) | 871 (35.1%) | 158 (11.9%) | 98 (28.8%) |
| Q3 (1,300-2,399) - n (%) | 3,388 (36.4%) | 919 (38.8%) | 1,254 (45.2%) | 998 (40.2%) | 93 (7.0%) | 124 (36.5%) |
| Q4 (>2,400) - n (%) | 720 (7.7%) | 393 (16.6%) | 197 (7.1%) | 84 (3.4%) | 9 (0.7%) | 37 (10.9%) |
| **Practice distance from an academic hospital (Kilometer)** |  |  |  |  |  |  |
| Greater (>10 km) - n (%) | 6,074 (65.3%) | 1,597 (67.4%) | 1,780 (64.1%) | 1,698 (68.4%) | 787 (59.1%) | 212 (62.4%) |
| 5-10 km - n (%) | 1,290 (13.9%) | 363 (15.3%) | 392 (14.1%) | 288 (11.6%) | 190 (14.3%) | 57 (16.8%) |
| Lower (5 km) - n (%) | 1,937 (20.8%) | 410 (17.3%) | 603 (21.7%) | 498 (20.0%) | 355 (26.7%) | 71 (20.9%) |
| **PCPs group size** |  |  |  |  |  |  |
| 1 physician - n (%) | 294 (3.2%) | 294 (3.2%) | 64 (2.7%) | 49 (1.8%) | 47 (1.9%) | 116 (8.7%) |
| 2 physicians - n (%) | 290 (3.1%) | 290 (3.1%) | 64 (2.7%) | 52 (1.9%) | 59 (2.4%) | 95 (7.1%) |
| 3-4 physicians - n (%) | 613 (6.6%) | 613 (6.6%) | 167 (7.0%) | 124 (4.5%) | 128 (5.2%) | 165 (12.4%) |
| >5 physicians - n (%) | 8,104 (87.1%) | 8,104 (87.1%) | 2,075 (87.6%) | 2,550 (91.9%) | 2,250 (90.6%) | 956 (71.8%) |
| **Total visits per rostered patient – mean (SD)** | 3.6 (1.9) | 4.29 (1.39) | 3.38 (0.84) | 3.11 (0.87) | 3.34 (4.04) | 4.50 (1.39) |

*Family Health Group where physicians are paid a mix of fee-for-service along with bonuses and premiums. Capitated non-Team includes models, i.e., Family Health Organization and Family Health Network where physicians are paid a mix of capitation payment, bonuses, premiums, and fee-for-service but they are not part of a Family Health Team (FHT). FHTs are interdisciplinary models of care, where physician can be paid through capitation with bonuses, premiums, and fee-for-service or salaried mechanisms. Solo FFS: Patients are not formally part of an enrolment model but receive care from a regular primary care physician who is paid purely fee-for-service. Other PEM models include smaller specialized patient enrolment models.

**Table 4.B:** Patients’ characteristics by practice model, Ontario, January 1, 2019, to December 31, 2019.

| **Patient Characteristics** | **Total Rostered Patients** | **Family Health Group** | **Capitated non-Team** | **Family Health Team** | **Solo FFS** | **Other PEM Models** |
| --- | --- | --- | --- | --- | --- | --- |
|  | N=11,810,698 | n=3,730,899 | n=3,902,404 | n=3,113,502 | n=587,449 | n=476,444 |
| **Patients age - Mean (SD)** | 41.05 (8.25) | 40.08 (22.06) | 43.35 (22.84) | 42.82 (23.54) | 38.07 (22.52) | 41.28 (22.31) |
| **Patients sex** |  |  |  |  |  |  |
| Female - n (%) | 6,140,036 (52.0%) | 1,917,534 (51.4%) | 2,052,562 (52.6%) | 1,644,990 (52.8%) | 289,641 (49.3%) | 235,309 (49.4%) |
| Male - n (%) | 5,670,662 (48.0%) | 1,813,365 (48.6%) | 1,849,842 (47.4%) | 1,468,512 (47.2%) | 297,808 (50.7%) | 241,135 (50.6%) |
| **Patients with twice the complexity relative to the average population**** |  |  |  |  |  |  |
| No - n (%) | 10,188,586 (86.3%) | 10,188,586 (86.3%) | 3,246,823 (87.0%) | 3,363,112 (86.2%) | 2,656,703 (85.3%) | 512,769 (87.3%) |
| Yes - n (%) | 1,622,112 (13.7%) | 1,622,112 (13.7%) | 484,076 (13.0%) | 539,292 (13.8%) | 456,799 (14.7%) | 74,680 (12.7%) |

**Based on the CIHI’s Population Grouping Methodology.
