# Supplementary material for: Association between primary care physicians’ practice models and referral rates to specialists: A sex-based cross-sectional study
Source: PLoS One. 2025 Apr 28;20(4):e0322175. doi: 10.1371/journal.pone.0322175 (PMC12036902; doi:10.1371/journal.pone.0322175)
Supplement: S5 Table — (DOCX) [file pone.0322175.s005.docx]

**S5 Table.** Unadjusted relative risk of specialist referrals, stratified by PCP’s sex, Ontario, January 1, 2019, to December 31, 2019.

| **Total referrals** | **Female PCPs – RR (95% CI)** | **Male PCPs – RR (95% CI)** |
| --- | --- | --- |
| **Patients age (mean)** | 1.03 (1.02-1.03) | 1.03 (1.03-1.03) |
| **Patients sex (% of rostered female patients)** | 1.01 (1.01-1.01) | 1.01 (1.00-1.01) |
| **Patients’ complexity (twice the complexity relative to the average population)** |  |  |
| No | (ref) | (ref) |
| Yes | 1.04 (1.04-1.04) | 1.05 (1.04-1.05) |
| **PCPs age – categories** |  |  |
| <40 yrs. (ref) | (ref) | (ref) |
| 40-49 yrs. | 1.03 (1.02-1.03) | 0.93 (0.92-0.93) |
| 50-59 yrs. | 0.97 (0.96-0.97) | 0.93 (0.93-0.94) |
| 60-69 yrs. | 0.98 (0.98-0.99) | 0.92 (0.92-0.93) |
| >70 yrs. | 0.91 (0.89-0.92) | 0.93 (0.92-0.93) |
| **Community size** |  |  |
| Large Urban | 0.96 (0.95-0.97) | 0.85 (0.85-0.86) |
| Medium Urban | 0.98 (0.97-0.99) | 0.95 (0.94-0.96) |
| Small Urban | 1.05 (1.04-1.06) | 1.05 (1.04-1.05) |
| Rural (ref) | (ref) | (ref) |
| Remote | 0.97 (0.96-0.98) | 0.91 (0.90-0.92) |
| Missing | 0.98 (0.97-1.00) | 0.92 (0.91-0.93) |
| **PCPs FTE (Quartile)** |  |  |
| Q1 (Equal or lower than 0.8766) (ref) | (ref) | (ref) |
| Q2 (0.8766 - 1) | 0.98 (0.98-0.99) | 1.13 (1.12-1.14) |
| Q3 (1 - 1.271) | 1.01 (1-1.01) | 1.15 (1.14-1.16) |
| Q4 (Equal or greater than 1.271) | 0.96 (0.95-0.96) | 1.17 (1.16-1.18) |
| **Roster size (Quartile)** |  |  |
| Q1 (<800) (ref) | (ref) | (ref) |
| Q2 (800-1,299) | 1.07 (1.07-1.08) | 1.26 (1.25-1.27) |
| Q3 (1,300-2,399) | 1.02 (1.01-1.03) | 1.20 (1.19-1.21) |
| Q4 (>2,400) - n (%) | 0.83 (0.83-0.84) | 1.08 (1.07-1.08) |
| **Practice distance from an academic hospital (Kilometer)** |  |  |
| Lower (5 km) (ref) | (ref) | (ref) |
| 5-10 km | 0.98 (0.98-0.99) | 0.94 (0.94-0.95) |
| Higher (>10) km | 0.93 (0.92-0.93) | 0.96 (0.96-0.96) |
| **PCPs group size** |  |  |
| 1 physician (ref) | (ref) | (ref) |
| 2 physicians | 1.22 (0.99-1.49) | 1.01 (0.81-1.26) |
| 3-4 physicians | 1.41 (1.17-1.69) | 1.24 (1.02-1.51) |
| 5+ physicians | 1.88 (1.58-2.23) | 1.55 (1.28-1.88) |
| **Practice model type^** |  |  |
| Family Health Team (ref) | (ref) | (ref) |
| Family Health Group | 0.88 (0.86-0.88) | 0.78 (0.78-0.79) |
| Capitated non-Team | 1.06 (1.06-1.07) | 0.97 (0.97-0.98) |
| Solo FFS | 0.72 (0.71-0.73) | 0.65 (0.64-0.65) |
| Other PEM models | 1.09 (1.08-1.10) | 0.78 (0.77-0.78) |
| ^ Family Health Group where physicians are paid a mix of fee-for-service along with bonuses and premiums. Capitated non-Team includes models, i.e., Family Health Organization and Family Health Network where physicians are paid a mix of capitation payment, bonuses, premiums, and fee-for-service but they are not part of a Family Health Team (FHT). FHTs are interdisciplinary models of care, where physician can be paid through capitation with bonuses, premiums, and fee-for-service or salaried mechanisms. Solo FFS: Patients are not formally part of an enrolment model but receive care from a regular primary care physician who is paid purely fee-for-service. Other PEM models include smaller specialized patient enrolment models. | | |
