# Supplementary material for: Association between primary care physicians’ practice models and referral rates to specialists: A sex-based cross-sectional study
Source: PLoS One. 2025 Apr 28;20(4):e0322175. doi: 10.1371/journal.pone.0322175 (PMC12036902; doi:10.1371/journal.pone.0322175)
Supplement: S6 File — (DOCX) [file pone.0322175.s006.docx]

**S6.** Brief description of Family Health Team (FHT).

Family Health Teams (FHTs) are not-for-profit primary healthcare organizations in Ontario, established in 2005 and designed based on the Patient Medical Home Model. They are the largest team-based care model in the province, serving 3.5 million patients. FHTs consist of primary care physicians, who are paid through separate agreements, and allied healthcare professionals.

FHTs focus on health promotion, disease prevention, and chronic disease management, including mental health, diabetes, and lung health. They cater to diverse populations, including rural, northern, and specialized patient groups with unique healthcare needs.

Only selected primary care physician payment models, such as Family Health Organizations (FHOs), Family Health Networks (FHNs), Blended Salaried Models (BSMs), and Rural and Northern Physician Group Agreements (RNGPAs), are eligible to join FHTs. These models compensate physicians through a blend of capitation, fee-for-service, bonuses, premiums, or salary.

However, not all FHOs, FHNs, or RNGPAs are affiliated with FHTs, as joining or expanding an FHT is typically based on a selective application process, with only successful groups receiving additional support.

Fee-for-service models (such as solo FFS, Comprehensive Care Model, and Family Health Groups) are not eligible to join the FHT model. While some smaller patient enrollment models may include allied healthcare professionals, their composition and structure differ significantly from FHTs, and they are also ineligible to join FHTs.

**References:**

Association of Family Health Teams of Ontario. Facts & Stats. Link: *https://www.afhto.ca/why-team-based-care/facts-stats*

Ontario Medical Association. 2018 FHT Funding Agreement. Link: *https://www.oma.org/practice-professional-support/starting-your-practice/primary-care-models/*

Government of Ontario. Family Health Teams. Link: *https://www.ontario.ca/page/family-health-teams*
