# Supplementary material for: Association between primary care physicians’ practice models and referral rates to specialists: A sex-based cross-sectional study
Source: PLoS One. 2025 Apr 28;20(4):e0322175. doi: 10.1371/journal.pone.0322175 (PMC12036902; doi:10.1371/journal.pone.0322175)
Supplement: S1 Fig — (DOCX) [file pone.0322175.s008.docx]

**S1 Fig.** Adjusted association of referrals to different specialties, stratified by PCP’s sex, Ontario, January 1, 2019, to December 31, 2019.

**
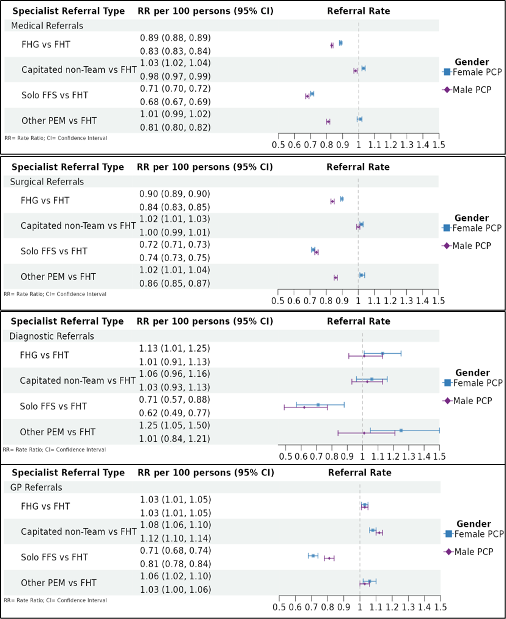
**Note: Adjusted for patients age (mean), patients sex (% of female patients), patient’s complexity twice of the average population, PCPs age, community size, PCPs FTE (quartile), roster size (quartile), practice distance from an academic, and PCPs group size.
